# A Treatise on Diseases of the Joints

**Published:** 1846-07

**Authors:** 


*Traité des Maladies des Articulations; accompagné d'un Atlas.* Par A. BONNET, Professeur de Clinique chirurgicale à l' Ecole de Médecine de Lyon, &c. *Paris,* 1845.

*A Treatise on Diseases of the Joints.* By A. BONNET, Professor of Clinical Surgery in the Medical School of Lyons, &c.—*Paris,* 1845. 2 vols. 8vo, pp. 1229.

IN our January Number (Brit. and For. Med. Rev., vol. xxi, p. 124) we gave an outline of MM. Bonnet and Richet's views respecting the general pathology of diseases of the joints, and we shall now proceed to examine M. Bonnet's doctrines as to the general etiology and general treatment of those affections.

The causes of diseases of the joints may be internal or external, and the most important of the former, on which M. Bonnet especially dwells, are those general or constitutional dispositions termed diatheses. The existence and character of a diathesis can only be known by its effects—its intimate nature is entirely unknown; but if we see fungosities, pus, or tubercles deposited (especially if they are so in several organs simultaneously), without any appreciable external cause, we infer that there is a disposition to such deposition, and that disposition is a diathesis. M. Bonnet, as we have seen, divides the morbid products in diseases of the joints into three classes: those which become perfectly organized—those whose organization is arrested—and those incapable of organization; and he chaterizes the diatheses that most commonly produce diseases of the joints according to their tendency to excite the deposition of one or other of those species of morbid products. The first is the *rheumatic* diathesis, the most favorable of all, in which there is a tendency to deposit plastic lymph; the second he terms the *fungous* diathesis, as it is characterized by the formation of "*fungosities;*" the *purulent*, the *tubercular*, and the *gouty* diathesis belong to the third class, being accompanied by the deposition of tubercle, pus, and uric acid, all incapable of organization. Often, however, there is no marked line of distinction between some of those diatheses. However distinctly marked in some cases, in others they may be mixed; *fungosities*, for example, are frequently infiltrated with pus, or may coexist with chronic abscess in some other situation. Indeed this liability to fusion has caused, M. Bonnet maintains, several really distinct constitutional conditions to be confounded under the common appellation of scrofula. Thus he considers the tubercular diathesis as different from scrofula; and this distinction made, he urges that among the other patients commonly termed scrofulous, individuals presenting very different general characters may be found. Some are pale, emaciated, with sallow cheeks, thin eyelids, lips, and septum nasi, and exhibit no trace of enlargement of the glands; these are the characters of the *purulent diathesis*, and they prevail in persons affected with chronic abscess. In others again the face is full, the eyelids, lips, and alæ nasi tumid, the glands of the neck usually large, and the complexion is commonly more or less florid, at least until the health has been undermined by the progress of disease. Such patients are usually liable to congestions with mucous secretions, their eyes are frequently red, the eyelashes adhering from a tenacious mucous secretion, slight causes excite obstinate catarrhs, and in children a crusted exudation often occupies the hairy scalp. Such are the signs of the scrofulous diathesis properly so called, which M. Bonnet prefers terming the "*fungous*" diathesis, as it is with this diathesis that "*fungosities*" most frequently coexist. (Vol. ii, pp. 25 et seq.) Still, as has been already said, those diatheses shade gradually into each other and often coexist; and this circumstance shows a close relation between them, which, M. Bonnet argues, is confirmed by other considerations. Thus their causes are all more or less common; they are all hereditary, and such is their connexion that parents affected with one diathesis may transmit either of the other to their children; their most frequent occasional causes are also the same, viz. chills, damps, suppression of the cutaneous secretion, eruptive fevers, &c. Their general symptoms also agree in this, that the functions of calorification, of the digestive organs, and of the skin, are deranged in each; they also frequently demand the same treatment, e. g. stimulating the skin, sulphureous and saline spas, iodine, alkalies, tonics, &c. Such points of agreement indicate that however widely different in other respects, something essential is common to all. What is it, then, that so greatly modifies this common term? To this M. Bonnet replies:

"I am inclined to believe that the same morbid principles introduced into the economy produce sometimes one, sometimes another, of these affections, solely because of the different predispositions which are peculiar to each individual and to each age ; children are predisposed to the secretion of pus and fungosities, they become scrofulous; youths and adults have a tendency to inflammations more rapid in their progress, they are predisposed to acute rheumatism; in middle and advanced life there is a tendency to maladies slow in their progress, and chronic rheumatism is of frequent occurrence. In my mind, scrofula and rheumatism have this in common, that they are caused by the same morbific principles, and their differences solely depend on the way in which each age and each constitution reacts under the influence of this cause." (Vol. i, p. 104.)

M. Bonnet founds his diagnosis, and consequently his prognosis, of *chronic* diseases of the joints mainly on the external characters indicating the existing diathesis. Thus, when fluctuation cannot be detected in a diseased joint, it is often impossible to determine by manual examination whether the fibrous or fungous tissue deposited in the joint is or is not infiltrated with pus; no doubt the more indurated the tumour the greater is the deposit of fibrous tissue and the more favorable the prognosis, while, on the contrary, the more yielding it is, the more do "*fungosities*" predominate; but in doubtful cases a tolerably accurate conclusion can only be arrived at by considering the general condition of the patient. Thus, if a chronic abscess coexists with a diseased joint of a doubtful character, we may certainly conclude that there is a tendency to the formation of a chronic abscess in the joint; while if fungosities exist elsewhere they are present in the joint also. But, above all, the general condition of the patient must be scrupulously examined—the countenance, complexion, &c., the capability of resisting fatigue and vicissitudes of temperature. If the signs of the purulent diathesis predominate, though "*fungosities*" only are deposited, they will probably become infiltrated with pus, while, if the scrofulous diathesis is well marked, fungosities, however soft, may escape suppuration, and become converted into fibrous tissue; and, finally, when the general signs of a good constitution are present a favorable issue may be anticipated, as organized matter has most probably been secreted. The same considerations are our only guide when there is no appreciable lesion of a joint in which the patient experiences some pain and some restraint of motion; in such a case it is only possible to say what will be the tendency of the disease should it become aggravated. If the chronic purulent diathesis exists those pains forebode a chronic abscess. If the scrofulous diathesis prevails fungosities are imminent. We have set forth M. Bonnet's views in this matter pretty fully, inasmuch as the following brief quotation shows the cardinal importance he attaches to them:

"If this work differs from those already published on diagnosis, it does so particularly in the care taken to estimate concomitant lesions and the general state of the constitution." (vol. i, p. 113.)

The two great external causes of diseases of the joints are cold, and especially damp. Their influence indeed is generally admitted; but M. Bonnet complains that the special conditions which render them innocent or injurious have not been determined, and this point he investigates at great length. As regards cold, M. Bonnet's doctrine is just this—1st, that the chill produced by cold air is much more injurious than that caused by cold water; and, 2d, that though the action of cold, especially cold air, is very dangerous when perspiration has been excited by bodily exertion, it is almost innocuous, and if the refrigerating medium is cold water completely innocuous, when passive perspiration exists, such, for example, as is produced by enveloping the body in flannel or other bad conductor of heat. This opinion M. Bonnet chiefly founds on the results of the hydropathic treatment, which he asserts has never been followed by inflammatory mischief in any case, including some hundreds in his own practice. Damp, however, is the most frequent agent that leads to diseases of the joints, and its influence is the more mischievous, as it acts by deteriorating the constitution. The vapour of pure water, or simple atmospheric moisture, is indeed, M. Bonnet thinks, quite harmless, but the moisture exhaled from walls recently built, frequently wet, or otherwise rendered damp, or derived from any similar source, is eminently injurious; producing in early life the fungous or purulent diathesis, or both, and generating rheumatism in adults. It is true that many under such circumstances apparently escape with impunity, but M. Bonnet maintains that their health is nevertheless almost uniformly really impaired. Thus the parents of a scrofulous child will often deny that the dampness of their dwelling has caused the disease, alleging that their own health is unimpaired though they inhabit the same house; but M. Bonnet has almost invariably found that their health is impaired, they are liable to catarrh, sore throat, ophthalmia, wandering pains, &c., on exposure to those transitions of temperature which are inseparable from the ordinary occupations of life. In support of these statements M. Bonnet enters into several details respecting the construction of the houses, and the habits of the manufacturing population in Lyons and the adjacent districts, to which we can only thus generally refer. (Vol. i, pp. 92 et seq.)

In the chapter on *general therapeutics,* M. Bonnet first discusses the local and then the general treatment; but we shall consider the latter subject first, as the chief indeed the only point therein requiring notice is directly connected with M. Bonnet's etiological views.

In constitutional diseases of the joints, according to M. Bonnet, the functions that are most frequently deranged are those of the skin. In many scrofulous and rheumatic patients those functions appear to be natural; but they are really disordered unless the perspiration is acid—possesses its peculiar smell—contains its normal salts—and unless the individual, when suitably clothed, sustains no sensation of cold in ordinary alterations of temperature. But all those conditions seldom exist in constitutional diseases of the joints, and, where they do not, our primary object should be to restore them; in effecting which, according to M. Bonnet, hydropathic baths are the remedy to which there is *nil simile nil secundum.*

We of course shall not describe the mode of administering those baths, as any of our readers who may not be acquainted with the process will find a full account of it in a former Number of this Journal. (No. XXVIII, p. 429.) But we shall briefly state the circumstances which M. Bonnet says specially indicate and contraindicate the practice, with the precautions he recommends in adopting it.

The hydropathic baths, then, are always indicated—when the perspiration is suppressed on the one hand, or is passive and copious on the other, and also when it has lost its odour—when the cutaneous circulation is languid—when the extremities are habitually cold, or when a sensation of coldness is experienced in or near the affected joint—when damp has been the exciting cause of the disease—when the pain, stiffness, &c., of the joint increase in winter and autumn, and diminish in summer, or generally are influenced by changes of weather—and, finally, when the patient is subject to wandering pains or transient shiverings. (Vol. i, pp. 167 and 553.) On the other hand, the treatment is contraindicated when the thorax, to use M. Bonnet's phrase, is *impressionable,* when there is habitual cough, and *a fortiori* when pulmonary tubercles exist; there is little hope of success when the patient is pale and much enfeebled, the favorable effects of the baths being the more decided the stronger and the greater the power of secretion of the individual. At first, if the patient is feeble the bath should be at the temperature of 18 or 20° C. (64 to 70° F.) and gradually lowered to 15°, 12°, and 10° C. (59, 53, 50° F.), and ultimately, when opportunity offers in winter, gradually lowered to 0° C. (32° F.) The time of immersion in the bath should be similarly graduated from two minutes to five or even ten minutes, which latter period should seldom be exceeded; but, irrespective of time, whenever the shock is severe, the patient shivering and his teeth chattering, he should be at once removed from the bath, as otherwise the action may be imperfect, or even entirely fail. M. Bonnet confirms the statements of others, that the perspiration during the first stage of the process has very frequently an odour *sui generis,* and, in scrofula especially, is often unsupportably fetid. Under the influence of the treatment the skin becomes soft and perspirable, the fetid perspiration, the coldness of the extremities, the sensitiveness to alternations of temperature, disappear, and the skin becomes more vascular, and assumes a healthy colour. From all this M. Bonnet thinks that there is no hypothesis involved in the conclusion that the hydropathic treatment acts by eliminating morbid principles, re-establishing natural perspiration, and invigorating the function of calorification. He is also quite satisfied that the injurious consequences popularly attributed to chills never follow the use of hydropathic baths, and accounts for this by the distinction he seeks to establish in his chapter on etiology between perspiration excited by exercise and that caused by warm clothing, and also between the chill produced by cold air and by cold water. We have already stated the general indications specified by M. Bonnet for the employment of hydropathic baths:—now for a few words respecting their applicability in particular diseases. M. Bonnet disapproves of them in acute inflammation, whether rheumatic or not; but he was induced to try the wet sheet in a few such cases, and without a favorable result. When, however, the malady is protracted, say beyond two months, and tends to become chronic, the fever having subsided, but swelling pain, stiffness, &c., remaining, then he has found the hydropathic treatment most efficacious. (Vol. i, pp. 369-73.) In the chapters on chronic arthritis and chronic rheumatism several cases thus treated are detailed. One is a case of chronic rheumatism of ten years' duration; there was hydarthrosis, with thickening of the soft parts of both knees and one wrist, displacement of the lower extremity of the ulna, nodosity of the joints of several fingers, and a similar condition of the joints of the tarsus. The patient suffered great pain, especially at night, and was unable to walk, the state of the hands preventing the use of crutches. The disease, moreover, had been contracted in the rainy season in the West Indies. After the administration of seventeen hydropathic baths it is reported that "the hydarthrosis of both knees, of the feet, and of the wrist is completely dissipated, the swelling of the fingers is scarcely perceptible ; the only remaining anatomical alteration is the displacement and mobility of the ulna, with slight doughiness of the adjacent soft parts. For the last fifteen days the pain and sense of coldness have entirely disappeared, the motion of the knees, feet, and wrist are restored, and any object can be firmly grasped with either hand." (Vol. i, pp. 497 et seq.) We have abstracted this case, as M. Bonnet says "the results are undoubtedly the most remarkable I have obtained from the use of cold baths in chronic rheumatism." (p. 531.) Notwithstanding this favorable result, however, M. Bonnet elsewhere says that in chronic arthritis and chronic rheumatism there is no legitimate hope of success unless the disease is recent, and the anatomical alterations slight (p. 416); and, again, that a complete cure is impossible if fibrous tissue is deposited within or external to the joint; that when serum is effused a cure is possible, but much more difficult than when pain and stiffness only are present. (p. 520.) In acute gout M. Bonnet, though he would not risk the bath, applied the "wet sheet" in one case with the most satisfactory result. (p. 562.) He has had no opportunity of treating chronic gout, but has no doubt that the hydropathic treatment in its full vigour would be eminently beneficial in it. (p. 569.) Hydropathic baths are strongly recommended in hydarthrosis, but no cases in which the treatment was employed are given. (p. 441.) In "*fungous tumours*" of the joint the same treatment is strenuously recommended as the most potent means of re-establishing the general health. In proof of its efficacy in modifying the scrofulous diathesis, M. Bonnet says that he has found it most successful in obstinate scrofulous ophthalmia, it being indeed the only general treatment by which he has cured the disease without the intervention of local applications. He admits that it is much less efficient when "*fungosities*" or pus exist in the joints, but even then he has always found the plan safe, and, despite a certain number of failures, he says, "I have recently seen two cases in which the success was very remarkable. The first was that of a child, aged 4, affected with a number of intermuscular abscesses in both legs, round the elbow, and in the neck." After three months' treatment a perfect cure was obtained. "The second case was that of a child, aged 3½, whose hands were covered with fistulæ communicating with the bones of the phalanges and carpus, which were necrosed and infiltrated with pus ;" there was a perfect recovery in four months. (Vol. ii, p. 34.) The baths are not recommended in the purulent or tubercular diathesis. And, finally, we are told that in chronic wandering pains, without any appreciable material alteration, provided the patient be not feeble, the plan "is of marvellous efficacy ; no method can be compared to it especially in removing susceptibility to cold." In this instance M. Bonnet says *probatum est*—as he derived the greatest advantage from the plan in his own proper person ; and he warns the patient not to be discouraged if he finds his sufferings increase at first, and to recollect that pains which have existed for years cannot be permanently cured without continuing the treatment from three to six months. (Vol. ii, p. 117.)

As to internal remedies in chronic diseases of the joints, M. Bonnet does not draw very largely on the Materia Medica. He thinks very favorably of sulphureous and saline mineral springs, both as baths and internally. (pp. 178-88.) Iodine he thinks may be useful when the "*fungous*" diathesis is well marked; but he deems it injurious when the patient is pale and thin; and its full utility in any case cannot be obtained, he is convinced, unless iodine baths, according to M. Lugol's method, are conjoined with its internal administration. Tonics he rejects, except special indications call for them. Iron he deems quite useless. Cod liver-oil he speaks favorably of, without specifying the cases he gives it in. (Vol. ii, pp. 26-32.)

*Local Treatment.* We have stated the great importance M. Bonnet attaches, in all diseases of the joints, to placing the limb in a suitable position, and keeping it motionless in that position during a certain period; the former object is effected by manual extension, or permanent extension by means of a suitable apparatus, with or without tenotomy, according to the circumstances of the case; permanent immobility of a limb cannot, M. Bonnet urges, be obtained by simply confining a patient to bed. Instrumental means must be resorted to, so contrived that part of the joint shall remain exposed, that no pressure shall be exerted on the joint, and that the posterior surface of the limb shall be supported. For this purpose M. Bonnet prefers hollow or cup splints, made of a trellis of iron wire, which combine the advantages of being cheap, light, cleanly, and easily made, and, above all, of being easily moulded into the shape suited to each particular joint. M. Bonnet gives a minute account of the mode of preparing and applying those splints to each joint, for which we must refer to his work and the accompanying plates. We have also already stated M. Bonnet's views as to when immobility should be replaced by passive motion of the joint (vol. i, pp. 124 et seq.); but we must however here refer to the *results* of this practice.

In a recent Number of this Review (No. XXXVI, pp. 359-60), we had occasion to advert to the question of straightening the knee-joint during the existence of active disease, which has been long practised by Lugol, Duval, and some other French surgeons, and we expressed our more than doubts of the safety of the proceeding. M. Bonnet has, we believe, carried this practice further than any other surgeon, inculcating it, in fact, as a rule without limitation or exception; and it will therefore be well to give the general results of the cases detailed in the present work in which the method was adopted. With respect to acute cases, M. Bonnet gives six cases in which the knee, three in which the hip, and one in which the ankle were affected with "very intense acute arthritis, in which the antiphlogistic treatment proved useless until the joint was brought into a suitable position, a proceeding which was immediately followed by immediate and permanent amendment." (Vol. i, pp. 313 et seq.; vol. ii, pp. 205-10, 356-61, 447-50.)

"Those cases," M. Bonnet says, in his observations on some of them, "show the extent to which the most powerful antiphlogistics and the means best suited to allay pain may be powerless in inflammation of the knee, so long as the patient is permitted to retain the vicious position he has adopted; and how greatly we relieve the patient and check the progress of a formidable inflammation by placing and firmly retaining the limb in a suitable posture." (Vol. ii, p. 208.)

It is not however pretended that the method is infallible, M. Bonnet goes on to say—

"In all the cases which I have detailed, the constitution of the patient was good, and the acute had not supervened on a chronic inflammation. In all those cases the amendment consequent on straightening the limb was immediate and permanent. But the result has been different with patients of deteriorated constitution, or who were antecedently affected with chronic inflammation of the joint; in the latter circumstances indeed, even where the constitution was good, though the treatment produced a rapid amendment, it did not effect a complete cure, which, if achieved at all, required a long time to accomplish." (Vol. ii, p. 210.)

As to the utility of the practice in chronic diseases of the joints, M. Bonnet seems to regard that as an admitted point, as he gives but one case as illustrative of its advantages; this is one of *fungous* tumour of the knee, and rectifying the position of the limb seems to have had a very beneficial effect. (Vol. ii, p. 227.)

M. Bonnet performed numerous experiments with the *actual cautery* and the *moxa*, which he regards as essentially different in their action from caustic, inasmuch as they cause a high degree of local excitement. This excitement he attributes to the heat communicated to the subjacent structures, and he examined the circumstances which influence the propagation of the heat. The most important of these is the integrity of the skin. The transcurrent cautery heats the tissues to the depth of about half an inch; but if the cautery is applied so as to destroy the skin, the heat scarcely passes beyond the slough in contact with the iron, both because the contact of moisture cools the iron, and chiefly because the skin, being a better conductor of heat than the other structures, its integrity favours the propagation of the heat. The heat of a large moxa slowly burned penetrates much deeper than that of the cauterising iron. Those considerations, M. Bonnet thinks, explain the aggravation of symptoms sometimes observed after the application of the cautery or moxa to diseased joints, before the complete subsidence of any intercurrent inflammation, which he considers especially liable to occur in a superficial joint, being scarcely ever observed in disease of the hip or of the spine. (Vol. i, pp. 148-62.) M. Bonnet has often employed the transcurrent cautery in Hydarthrosis, and thinks its efficacy is greatly overrated; he has always seen the quantity of serum greatly diminished by its use, but it never completely disappeared, and the motions of the joint remained as much or even more embarrassed than previously. (Vol. i, p. 446.) "*Fungosity*" of the joints is the disease in which the cautery is generally indicated, and here M. Bonnet prefers the moxa, which he deems the most efficacious local means at our command in this affection. Excepting only placing the limb in a good position, and the judicious alternation of rest and motion, little is to be expected from a few applications of the moxa; it must be employed with energy and perseverance to do good, and in the tolerably numerous cases in which M. Bonnet has seen the moxa effect a slow but complete resolution, the whole anterior surface of the joint had been burned in succession; the moxa, too, he considers should be much larger than those usually employed. (Vol. ii, p. 48.) In the chapter on chronic abscess of the joints, M. Bonnet incidentally states that in chronic abscess of the cellular tissue, the best practice unquestionably is to destroy the entire surface of the abscess with caustic or the cautery; and in the chapter on diseases of the hip-joint, we find four cases of large intermuscular abscesses in which this practice was pursued, and they are certainly sufficiently remarkable to demand notice. The first is the case of a girl aged 21, who had been for seven months affected with an abscess which occupied the entire outer aspect of the hip, and which it was presumed did not communicate with the joint. The abscess lay so deep that three successive applications of potassa fusa c. calce failed to open it, and it was then laid open by a vertical incision, 7½ inches long, and 1½ inch deep, made with the metallic cautery. A quantity of thin serous pus escaped, and on exploring the cavity, the finger was found to pass behind the great trochanter and neck of the femur. M. Bonnet now concluded that the abscess did communicate with the hip, notwithstanding the absence of symptoms to that effect, and hesitated for some time, but at length persevered in his original intention, and *extinguished* twelve cauterizing irons in the abscess so as to destroy its entire surface, including the sinus leading behind the neck of the femur. On the third day violent inflammation set in, which promptly subsided on the fifth day, after the action of an emetic. By the twelfth day the separation of the sloughs left a clean granulating surface, and in three months the patient recovered, with perfect use of the limb. Three other very similar and equally successful cases are detailed, in one of which the abscess was even larger than in the foregoing case. There is likewise a fifth case, in which M. Bonnet (though he elsewhere repeatedly says the practice is inapplicable when an abscess communicates with a joint), having to deal with an abscess connected with the hip, which had opened on the front of the thigh, cut away the undermined integuments to the extent of a hand's breadth, and applied chloride of zinc to the whole surface of the abscess, and to the fistulous passage leading to the joint; the condition of the patient, it is said, became much improved, but it does not very accurately appear to what extent. (Vol. ii, pp. 379-86.) M. Bonnet was assuredly very fortunate in the issue of those cases, but for our part, we should be slow to tempt fortune by following his example.

M. Bonnet attributes the mischief that so usually follows the opening of a joint, or of a large chronic abscess, to the admission of air,—not that he thinks air exerts any irritant action, but he attributes its bad effects entirely to its enabling the fluids (whether synovia, blood, or pus) which it comes in contact with to putrefy; and the decomposition of the liquids, he maintains, always precedes and accompanies the bad symptoms, the discharge becoming fetid and charged with air; so that the reality and progress of the putrefaction can be traced with test paper, showing that sometimes ammonia alone, and sometimes hydrosulphuret of ammonia is evolved. (Vol. i, pp. 24 and 263.) This decomposition leads to what M. Bonnet calls *putrid absorption*, in contradistinction to *purulent* absorption—the former characterized by the putrid discharge, by occurring at a later period, by being accompanied with intense continued fever, with the characteristic symptoms of extreme fetor of the stools and delirium, and by no internal abscesses being found after death; while the latter affection is ushered in by rigors, assumes a remittent form, is never accompanied with delirium, and abscesses are disseminated in various organs. (Vol. i, p. 259.) It is to avert this *putrid* absorption that M. Bonnet cauterizes the surface of a chronic abscess; the surface thereby ceases to be an absorbing one, and on the detachment of the sloughs there is a healthy granulating surface disposed to heal, while *purulent* absorption is not to be apprehended—as, according to M. Bonnet, it never (?) follows the application of either the actual or potential cautery. (Vol. i, p. 381.) Our author is so persuaded that such is the fact, that he has adopted the practice of cauterizing the surface of a wound whenever purulent absorption appears imminent, preferring chloride of zinc for this purpose, as it alarms the patient less, and dries the surface of the wound more thoroughly than the actual cautery.

> "I apply," says M. Bonnet, "this method especially in wounds, the result of cancers of the breast, &c. . . . So convinced am I from experience of the prompt and evident utility of the practice, that when after an operation I see the wound ash-coloured, yielding a serous or fetid discharge, with the swollen painful margin foreboding phlegmonous erysipelas, there being also burning fever and thirst, I unhesitatingly cauterize the entire surface of the wound. Amputations excepted, I have not yet met with one case in which the mischief was not arrested by the practice, which more than any other circumstance proves the power of the cautery to limit and localize the lesion." (Vol. i, p. 385.)

In the case of joints, however, M. Bonnet admits that the cautery or caustic is inapplicable, for reasons too obvious to require being detailed. How, then, are we to proceed in cases of obstinate hydarthrosis, or chronic abscess of joints? Evacuating the fluid by a simple puncture is safe but inefficient. Subcutaneous section of the synovial membrane would in hydarthrosis be quite safe, and, M. Bonnet is inclined to think, very probably efficient, as he saw one obstinate case of the affection cured by a fall which ruptured the synovial membrane of the knee, and diffused the fluid into the cellular tissue; but still he says he has never adopted the practice. (Vol. i, p. 453). By the way, we find he did; for we may here mention that M. Bonnet failed in attempting to repeat M. Goyraud's operation for extracting a loose cartilage from the knee-joint by the subcutaneous method; for he found it impossible to push the foreign body into the cellular tissue, though the abundant escape of synovia evinced that the synovial membrane was freely divided; after the operation, a previously existing hydrops articuli returned, which however the presence of the foreign body perhaps sufficiently accounts for. (Vol. i, p. 488.) Neither has M. Bonnet tried M. J. Guerin's proposal of evacuating the liquid, whether synovia or pus, by means of an exhausting syringe, being convinced that the liquid would again collect, as its mere evacuation would not modify the surface that secreted it, and the method he has adopted to attain this latter object is the employment of *irritant injections.*

The use of *iodine injections* in the treatment of hydarthrosis, serous cysts, chronic abscesses, &c. has latterly been warmly recommended by several French surgeons. M. Velpeau lays claim to having first applied the practice to the treatment of hydarthrosis, but M. Bonnet vindicates the priority in this particular for himself, as in March, 1841, he injected the knee-joint with iodine, and, encouraged by the success of this first attempt, repeated the operation ten times within the year in cases of hydarthrosis and abscess of the knee. An account of some of those operations was published in May, 1842, whereas M. Velpeau did not communicate the result of his first two cases to the Academy of Sciences until the 8th October, 1842, in which communication it was stated that he had injected the knee with iodine in July, 1839. The details of those two cases have been since published by M. Velpeau, in his '*Recherches sur les Cavités closes,*' whence it appears that the injection was in each of them thrown, not into the joint, but into synovial cysts on the inside of the leg, probably formed by the sheaths of the tendons constituting the "*Patte d'oie.*" M. Velpeau, however, concludes that the injection did enter the joint because of the inflammation of the whole articulation which followed. M. Bonnet urges that even if the fact was so, it occurred accidentally, and that the priority remains with him, but fully admits that the idea of the operation was suggested to him by the general principles for the treatment of serous accumulations contended for by M. Velpeau. So much for the question of priority; but it is more important to inquire what is the value of the matter in dispute; as a preliminary to which, the method itself must be described, and the origin of the practice noticed. It was in hydrocele that M. Velpeau first employed iodine injections; for deeming the wine injection, though very efficacious, too irritating, and seeing that it caused gangrene if accidentally thrown into the cellular tissue, he sought for some safer substance, and at length selected diluted tincture of iodine, being it would seem not aware, or at all events having forgotten, that, first, Mr. Martin, of Calcutta, and subsequently Dr. Oppenheim, of Hamburg, and several others, had anticipated him in the practice. M. Velpeau imagined that the resolvent action of iodine thus locally applied might be beneficial, as serous collections frequently depend on, or are connected with, congestion, hypertrophy, &c. of some adjacent organ, and he therefore sought to determine—1st, if tincture of iodine excited adhesive inflammation in close cavities; 2d, if it dispelled the chronic alterations which sometimes complicate dropsy of those cavities; and 3d, if, like wine, it caused gangrene when infiltrated into the cellular tissue. The first two questions he answers affirmatively, from the permanent cure of some hundreds of cases of hydrocele, accompanied with the almost constant disappearance of the hypertrophy of the testicle, which not unfrequently accompanies that disease. The third question he answers in the negative, because, on injecting tincture of iodine into the cellular tissue of animals, he never found it excite gangrene or even suppuration; and in seven or eight cases in which the injection accidentally escaped into the cellular tissue in the human subject, it was either harmless, or merely caused very limited suppuration. M. Velpeau being persuaded that tincture of iodine produces in close cavities purely adhesive inflammation, which scarcely extends beyond the point of the cavity it comes actually in contact with, was emboldened to extend its application from simple hydrocele to congenital hydrocele, and hydrocele of the hernial sac; and even in three instances attempted the radical cure of hernia by this method, pressure during all those latter operations being made at the inguinal ring to prevent the injection passing into the abdomen. In the two former of those affections he succeeded, but failed in the latter, the difficulty of injecting a simple hernial sac being such, that in his first attempt he thinks the whole of the injection was thrown into the cellular tissue, whence copious suppuration ensued; and though in the second and third cases he succeeded in obliterating the sac without the occurrence of the slightest unpleasant symptom, the hernia returned after two months. M. Velpeau also employed the iodine injection with perfect success and safety in two cases of serous cysts in the iliac fossa—in an encysted sero-sanguineous collection behind the uterus, ascending to the right iliac fossa; in a serous collection situate between the triceps and the femur, five inches long, by four in breadth; in two serous cysts of the axilla, three of the female breast, five of the neck, an unspecified number of similar tumours of the thigh, groin, and parietes of the abdomen, and also in four cases of encysted goitre; several of those various tumours being upwards of double the size of the clenched hand, and one as large as an ostrich egg. He further employed the injection in two *vast* purulent collections, one subclavicular, the other substernal; in the latter case with success, but in the former the tumour enlarged, the skin inflamed, and the cavity had to be laid open with the knife. Except in this last-mentioned instance, no unpleasant symptoms occurred in any of the foregoing cases, save in one case of encysted goitre, into which 100 grammes of the iodine injection were thrown (gramme = 15·444 grs.); on the sixth day there was high fever, and on the tenth an icteric tinge appeared on the face, neck, and greater part of the body, the fever gradually subsided, and the goitre was permanently cured. The icteric tinge was not exactly similar to that of jaundice, and M. Velpeau thence thinks it not impossible that it arose from absorption of iodine. Similarly favorable results were obtained with the various bursal cavities, except that the operation failed to effect a cure in about one half the cases of bursal tumours over the patella and the olecranon, a failure which M. Velpeau attributes to the inertia, low vascularity, and denseness of the structures in those regions. The next step was to try the method in tumours of the synovial sheaths of the tendons, in which it proved equally successful, when the tumour was unilocular and contained serum only, unmixed with fibrinous concretions. Thus M. Velpeau succeeded in several ganglions on the back of the hand and of the wrist, in two synovial cysts of the ham, each as large as a goose egg, in one between the tendon of the biceps and the radius, and in the case of the synovial sheath of the tibialis posticus; while he failed in a case of hour-glass tumour, containing hordeiform concretions, which extended from the palm of the hand beneath the annular ligament some way above the wrist. M. Velpeau now seriously entertained the idea of applying the iodine injection to the treatment of hydarthrosis, but was restrained, not so much from the dread of suppurative inflammation occurring, as from the apprehension of exciting adhesion of the synovial membrane, and thereby impeding the functions of the joint; but as this did not occur in the cases already referred to, in which M. Velpeau concluded that he had accidentally injected the knee while operating on a synovial tumour, which was supposed not to communicate with the articulation, he injected the knee directly in a series of cases, which we shall examine, together with those of M. Bonnet and other practitioners. (Recherches sur les Cavités closes, &c. pp. 112-65.)

To inject the knee-joint the leg must be extended. M. Bonnet pinches up a fold of the skin and passes in a hydrocele trocar at the base of this fold, in order that when the operation is complete the external and internal puncture shall not correspond, and thereby admit air. M. Bonnet also always holds the trocar as vertically as is consistent with the escape of the fluid, as air can scarcely enter while the trocar is full of liquid. M. Velpeau neglects these precautions, as he is satisfied by experience that the admission of a few bubbles of air is harmless. Both agree that it is not of much consequence at what point the joint is perforated, but prefer the upper and outer part of the joint, or rather whatever point fluctuation may be most evident at. M. Bonnet seems to use an ordinary trocar; M. Velpeau attaches great importance to employing an instrument not larger at the utmost than a hydrocele trocar. M. Bonnet merely allows six or eight drachms of synovia to escape, a quantity, in fact, equal to the bulk of the injection he throws in; M. Velpeau, on the contrary, empties the joint as completely as he can. M. Bonnet in almost every case injected pure tincture of iodine, calculating on its being diluted by the fluid left in the joint; M. Velpeau injects tincture of iodine diluted, it is not precisely said to what extent, but it would seem with from two to four parts of water. M. Bonnet allows either the entire or the greater part of the fluid to remain in the joint after the injection has been performed; M. Velpeau, on the contrary, seems to allow the whole of the injection to escape—we say *seems,* for there is great want of precision in many of M. Velpeau's statements. M. Bonnet would in future prefer M. Velpeau's method, as it excites greatly less inflammation than occurred in his own cases.

Ten cases of the operation are given by M. Velpeau in the work already quoted; but as two of them are doubtful, eight patients and nine operations remain, one patient having had hydarthrosis of both knees. We further find, from the Gazette des Hôpitaux de Paris, and Gazette Médicale de Paris (Sept. Oct. and Dec. 1845, and Jan. 1846), that he has since performed other operations, amounting to about fifteen in all, and also that M. A. Bérard has operated five times on two patients, and M. Robert once. M. Bonnet's operations are twenty-four in number—ten for hydarthrosis of the knee, performed on seven patients, both joints having been affected in three cases, and the same joint having been twice injected in another; and thirteen injections, some with alcohol and brandy, were practised in five cases of chronic abscess of the knee, six times within the space of three months in one case, three times in a second, and twice in a third. Finally, we may add that M. Jules Roux, of Toulon, operated on a large hydarthrosis of the shoulder-joint, which contained 500 grammes of fluid. (Bulletin de l'Acad. Roy. de Méd., 15th Jan. 1846.)

In all the cases which are detailed with any approach to precision, it appears that the operation was followed within a few hours by inflammation of the joint and fever; and in almost every case considerable, sometimes severe, pain was experienced at the moment of the operation. It is, however, very remarkable that in the great majority of cases the inflammation was moderate, though in some it assumed a very formidable character. Thus M. Bonnet, who writes with most praiseworthy candour, records some cases in the 'Bulletin de Thérapeutique,' t. xxiii, Nov. Dec. 1842, which are only alluded to in the present work, and we there find the following statements :—

"The inflammation ran on with alarming rapidity and intensity. The patient shrieked with pain during the entire day. Forty leeches were applied in the evening without giving any relief. The swelling, which was much greater than before the operation, was rapidly increasing. The tension of the skin was extreme. At seven in the evening, alarmed at the rapid increase of the swelling, I could devise no other expedient to restrain it than again plunging the trocar into the joint." And in another case, "The inflammatory reaction was intense; during the night there was fever, restlessness, and even commencing delirium. The joint swelled, and the skin was red, tense, and burning; nausea and vomiting set in, and those symptoms lasted three days."

The only instance in which suppuration occurred was in M. J. Roux's case of hydarthrosis of the shoulder; and though the patient ultimately did well, the case sufficiently shows, together with those just quoted, how utterly unfounded is M. Velpeau's quiet assumption that "the safety of iodine injections performed according to certain rules is now beyond dispute." Indeed, during the academic discussion on M. Velpeau's report on M. J. Roux's case, enough transpired to show that an iodine injection, like any other stimulating injection, should be employed with great caution, and that it does not enjoy the peculiar mildness of action attributed to it by M. Velpeau. According to M. Velpeau, an iodine injection, if infiltrated into the cellular tissue, never excites gangrene, and when thrown into a close cavity never produces suppurative inflammation—two propositions which he founds on surgical experience, and on experiments performed on animals by himself and others. But during the discussion in question (to be found in the journals already referred to), it appeared that MM. Bavot and Bouley had seen suppuration, gangrene, and death caused by injecting iodine into the cellular tissue and joints of animals; that gangrene of the scrotum had occurred after the injection of iodine in a patient of M. Jobert's, and a patient in the Hospital St. Louis; while a third case is published in a recent number of the 'Clinique de Montpellier.' The assertion that the iodine injection never causes suppuration in serous cysts is already disproved by M. J. Roux's case; and, moreover, suppuration and death ensued from injecting a cyst on the neck of a patient in the Hospital la Charité. It appears, then, that the iodine injection may produce precisely the same mischiefs that may follow the employment of wine and other stimulant injections proposed to be superseded by it; and we fully agree with MM. Blandin, Roux, and others, that though no mischief is yet known to have occurred from injecting it into the joints, disastrous results must inevitably follow from perseverance in the practice. There are few proceedings in medicine or surgery, however hazardous, which cannot be supported by an array of successful cases; and we would just as soon infer the safety of injecting the knee-joint in hydrathrosis from the cases we have enumerated, as we would infer the safety of passing a seton through the knee in the same disease from the result of ten cases reported to have been so treated with success by Dr. Müller. (Gaz. des Hôp. June 1842.)

But, supposing the practice safe, what is its efficacy? We must premise that, as regards hydarthrosis, the cases are, for the most part, so loosely described that it is often difficult to determine their nature; but it is yet quite clear that many of them were not cases of simple hydrops articuli, but that some more serious chronic alteration coexisted with the serous accumulation. Two only of M. Bonnet's cases terminated in a perfect cure; but M. Bonnet candidly says: "Both these patients were young (16 and 18), and in both the hydarthrosis was recent, having existed but eight days in one, and not quite three months in the other case. There was no crepitation in the joint, nor any tumefaction of the superficial soft parts." (Vol. i, p. 464.) In his third and fourth cases some stiffness of the joint remained, notwithstanding the employment of various accessory treatment. In the fifth case, the motions of the joint were somewhat *more impeded* after the operation. We have an accurate account of nine only of M. Velpeau's cases; in three the operation failed to effect a cure. We are not told the precise condition of the joint when the other six were pronounced to be cured; but only three recovered quickly, and those were simple uncomplicated cases, of recent date, in young healthy patients. We may here remark that one of these six patients died of fever three months after the operation, and the cartilage of incrustation was found thin and somewhat eroded, and the reticular structure of the condyles of the femur was highly vascular; death was, however, clearly unconnected with the operation. The three cases operated on by MM. Bérard and Robert were neither benefited nor injured; the disease, whatever it was, ran on its course, and amputation in the three cases was performed at a remote period.

It is useless to comment on these facts, beyond the simple inference that the operation cured those cases, some of which would certainly, and others probably, have recovered under the usual methods of treatment, and failed, in like manner, where those methods would have failed. We cannot, however, avoid noticing an amazing discrepancy between the precepts and practice of M. Bonnet and M. Velpeau. They both urge that blisters, moxas, &c., should be "*exhausted*" before recurring to injection in the treatment of hydarthrosis. M. Velpeau, in particular, dwells upon the efficacy of "*monster*" blisters enveloping the entire circumference of the joints, and mentions some apparently intractable cases in which he found his plan succeed; yet we find M. Velpeau adopting the injection in the first instance in almost, if not all, his latter cases; and M. Bonnet actually resorted to it in simple hydarthrosis of eight days' standing! We are not in possession of materials to form an opinion founded on facts respecting the value of iodine and similar injections in chronic abscess, but, from the results of the methods more or less analogous which have been tried, we would anticipate that it is a hazardous proceeding. The instances in which M. Bonnet has adopted this practice are but few. In one case of chronic abscess, double the size of the clenched hand, situated over the scapula, he evacuated half the pus and replaced it—not, indeed, with iodine injection, but with camphorated spirit of wine, which was left *in situ*. As no effect was produced, the injection was repeated three days after; violent inflammation, severe pain, and high fever now ensued, and to relieve the patient's sufferings caustic was applied to the tumour, where it seemed most disposed to open; in about six weeks recovery was complete. In the cases of chronic abscess of the knee on which M. Bonnet operated, the tumour partly consisted of fungous and lardaceous tissues; to use M. Bonnet's language, the "*fungous*" coexisted with the purulent diathesis, and three of the patients were of the respective ages of two, seven, and nine years. In one case alcohol, first, and three months after, undiluted tincture of iodine was injected. We are merely told that the reaction was very trifling, and that the patient left the hospital in a very satisfactory condition; as the joint contained no liquid, was exempt from pain, and its motions free. In a second case undiluted alcohol was injected, which caused scarcely any reaction; after thirty-four days the injection was repeated, very slight inflammation followed, and in three weeks some slight stiffness of the knee alone remained. In the third the abscess had existed for a long time and was very large, extending to the outer side of the joint and under the triceps to the middle of the thigh. Six irritant injections, the two first tincture of iodine, the third brandy saturated with camphor, were practised in the space of three months; the latter injection excited some reaction, the puncture opened, pus escaped freely, and the cavity under the triceps became gradually obliterated, but the joint continued to secrete pus copiously. A fourth injection (tincture of iodine) producing no effect, nine days subsequently the joint was injected with the still more stimulant liquid Fioraventi's balsam, which caused moderate inflammation without fever. The suppuration now gradually lessened, increased for some days after another injection of tincture of iodine, then again diminished, and in a fortnight the last fistula closed, the joint being free from liquid, and merely somewhat stiff. The fourth case only differs from the foregoing in that the injection (tincture of iodine three times repeated) caused severe pain; and though the patient left the hospital with an useful limb, the disease returned some months afterwards, in consequence of an injury of the joint, and the limb was amputated. The fifth patient was a woman, aged 28, who died of peritonitis fourteen days after the knee was injected with tincture of iodine. On dissection there was no trace of inflammation of the synovial membrane. In this case M. Bonnet says the tincture of iodine seemed almost inert; and he finally condemns the operation as useless in adults, restricting it to young patients whose constitutions are not too much deteriorated. (Vol. ii, pp. 84-96.) Since the publication of M. Bonnet's work, several other surgeons have employed iodine injections in the treatment of chronic abscesses, but we have not met with the particulars of their cases. We find it, however, generally stated (Gaz. des Hôp., Dec. 1845) that M. Jobert has cured several large chronic abscesses by this method, one being an "*enormous*" chronic abscess on the cervico-dorsal region, so large that little expectations of recovery were entertained. M. Velpeau's two cases already indicated are not given in any detail; suppuration, however, occurred in one of them. With respect to serous and other analogous collections, the occasional, perhaps frequent, efficacy and safety of iodine injections, cannot be disputed, but we are strongly convinced that their application in large accumulations of the kind is attended with great risk. We have already adverted to some facts which sufficiently bear out this conviction; and we may here observe that M. Bonnet's case of chronic abscess over the scapula shows how utterly unable we are to predict confidently what the effect of an irritant injection in a given case shall be, camphorated spirit having in the same patient at one time proved inert, at another having excited severe inflammation.

We have entered rather fully into the question of iodine injections, as they are now very extensively used on the Continent, and the confidence with which M. Velpeau and others proclaim their constant safety and almost constant success, is calculated to induce others to imitate their boldness. We may here mention, as illustrations, how indiscriminately these injections are employed by some, that Dr. Suytgaerens, of Puers, after performing paracentesis of the thorax in a case of empyema, repeatedly injected the pleura with an iodine injection, the result, we must add, was favorable, and the patient recovered. (Gaz. des Hôp., Fevr. 1845). More recently, M. Dieulafoy, of Toulouse, in a case of ascites, threw a quantity of iodine injection into the peritoneum, and, after diffusing it over the entire cavity, drew off about half the quantity of fluid injected. Febrile reaction, with slight pain in the abdomen, followed; cataplasms and mercurial frictions were directed. A month after, about half the cavity of the peritoneum seemed obliterated, but the fluid, having again collected superiorly, the injection was repeated with similar consequences. In about six weeks a small quantity of serum had accumulated, forming a circumscribed globular tumour; a third injection produced the same febrile and inflammatory symptoms. The ascites now finally disappeared, and was replaced by general anasarca, which yielded to purgatives. The patient ultimately recovered after a long convalescence, and experienced no inconvenience, save a dragging sensation in the abdomen on assuming the erect position. Finally, to conclude this subject, it may be noticed that, during the academic discussion so often referred to, it was suggested by MM. Laugier and Caventou, that the stimulant effects of diluted tincture of iodine was solely due to the alcohol it contained, and that the iodine was, so to say, mere surplusage. It may be, and we incline to the opinion, that there is nothing special in the local action of iodine, but unquestionably it powerfully contributes to the stimulant properties of those injections, as might be inferred from its action on the skin, and as is directly proved by the fact, that M. Bonnet has sometimes employed an injection composed of iodine, iodide of potassium, and water, and found that it excited active inflammation.

We have now noticed pretty fully whatever seems most peculiar in the general methods of treatment recommended by M. Bonnet, and what we have said respecting their application in particular cases precludes the necessity of examining that part of his work which treats of the diseases of each particular joint, especially as the author for the most part confines himself to the exposition of his own views, which have, we trust, been already sufficiently explained. A few points, however, require notice.

More doubt and difficulty occasionally arises in pronouncing an accurate diagnosis in certain cases of disease of the hip than occurs in affections of perhaps any other joint. Thus, the apparently simple questions of determining whether the affected limb is really or apparently longer or shorter than its fellow? and, in either case, what is the cause and symptomatic value of the phenomenon?—have received the most contradictory answers from writers of acknowledged eminence. The investigations of MM. Bonnet, Maisonneuve, and Parise, especially of the latter (Archiv. Gén. de Méd., July—Aug. 1843), have done much towards elucidating this difficulty.

Sir B. Brodie particularly pointed out the influence of lateral inclination of the pelvis in producing elongation, and occasionally also shortening, of the limb in morbus coxæ, according as the pelvis is elevated or depressed towards the affected side; and, since his observations, many, probably most, surgeons admit that the elongation is always, and the shortening frequently, apparent. M. Malgaigne next observed the curious fact that, if one side of the pelvis is strongly depressed, the lower extremities being perfectly parallel, both inspection and measurement then indicate a difference of eight or nine lines between the length of the two limbs—the limb corresponding to the depressed side of the pelvis *appearing longer to the eye,* but seeming *shorter* by *measurement* than its fellow, while the opposite limb, on the contrary, though the *shorter to the eye,* seems the *longer on measurement.* M. Malgaigne attributes this entirely to the lateral inclination of the pelvis approximating the crest of the ilium and the great trochanter on one side, and separating them on the other; while M. J. Guerin says this is but a particular case of the general rule, that any obliquity of the thighs to the axis of the pelvis modifies the apparent length of the lower extremities, whether determined by inspection or by measurement. Fricke also observed that the apparently elongated limb was found to be shorter than its fellow on measuring from the iliac spine to the outer malleolus, and mistaking this for real shortening. He attributed it to spasmodic action of the muscles, compressing the head of the femur into the cotyloid cavity. Such seems to have been the state of the question when MM. Bonnet and Parise commenced their inquiries.

MM. Bonnet and Parise both admit two varieties of elongation and shortening of the limb in morbus coxæ—*apparent* lengthening and shortening, and *real* lengthening and shortening—the former entirely produced by the position of the limb in relation to the pelvis, the latter depending on various causes. And first as to *apparent* variations of length.

The axis of the cotyloid cavity looks downwards, outwards, and slightly forwards, and the head of the femur, during every motion of the thigh, may be considered to revolve on an imaginary axis, antero-posterior in adduction and abduction, transverse in flexion and extension, parallel to the axis of the limb in rotation, and constantly changing during circumduction. All these axes traverse a common point, the centre of motion, which is *quam proxime* the centre of the head of the femur, and therefore of the cotyloid cavity. The distance between the centre of motion and the anterior iliac spine is invariable; but if lines are drawn from the centre of motion to the anterior iliac spine and to the external malleolus, those two lines move on each other like the legs of a compass, and, as they approach or recede from each other, their extremities approximate or separate. It is obvious, then, why measurements from the spine of the ilium to the malleolus vary with the position of the limb; and M. Parise's experiments, which seem to have been most accurately conducted, show—1st. That adduction increases and abduction lessens the length of the limb as *measured* from the anterior superior spinous process of the ilium to the malleolus. 2d. That the maximum elongation occurs during abduction combined with extension, as, on complete extension of the lower extremity, the iliac spine, the centre of the hip, and the outer malleolus, are in the same line. 3d. That abduction with flexion causes the greatest shortening. 4th. That, on measuring from the *posterior* superior spinous process of the ilium, the length of the limb also varies with its position, but inversely to the variations in the foregoing cases; for, as the posterior spinous process lies on a plane posterior, superior and internal to the centre of the hip-joint, the line joining those two points, runs upwards, backwards. and inwards, or in the reverse direction to a line joining the centre of the hip and the anterior iliac spine; whence the position which gives the greatest length on measuring from one of those points gives the least on measuring from the other.

Admitting that apparent difference of length is caused by the position of the two limbs being different in relation to the vertical axis of the pelvis, it would seem very easy to restore symmetry, and so remove the cause of error; and so it is when the disease is recent; but after some time we generally can only bring the trunk and the lower extremities into the same vertical line, and a deviation of the pelvis still exists, which cannot be removed, and the limbs are examined while really unsymmetrical with the axis of the pelvis. What, now, is the cause of the deviation of the pelvis?

MM. Bonnet and Parise both dispute Brodie's well-known explanation of the lateral depression of the pelvis towards the affected side in the early stage of morbus coxæ; for, were it true, the phenomenon should not occur, as it does, in cases where the patient has been confined to bed, and M. Parise maintains that when the pelvis is depressed on the diseased side, the position, so far from being selected by the patient as most commodious and least painful during standing and walking, is a false position and assumed from necessity, though the least commodious. M. Bonnet proposes the explanation, that patients in the early stage of morbus coxæ lie on the affected side, in which position the thigh is flexed on the pelvis and somewhat abducted, while, at a more advanced stage, pressure on the joint being painful. they lie on the sound side, whence the diseased limb is flexed, adducted, and rotated inwards. This amounts to saying that the pelvis is really not deviated, but that the limb makes an angle with the pelvis, and not the pelvis an angle with the limb. M. Parise's explanation is also based on this latter principle, but his exposition of its causation and operation is more ingenious and more consonant with facts than that of M. Bonnet. He maintains that the inclination of the pelvis is always subordinate to the position of the femur, and thus traces the influence of the position of the limb in the several stages of hip-joint disease. In the first stage the limb is almost constantly abducted, flexed on the pelvis, and slightly rotated outwards. The cause of this position is, M. Parise maintains, the presence of more or less fluid in the capsule of the hip-joint, for analogy and observation show that one of the first effects of the disease is to cause accumulation of more or less synovia in the joint, and M. Bonnet's experiments on injecting joints, repeated and confirmed by MM. Parise and Maisonneuve, prove that distension of the capsule of the hip with liquid causes the thigh to assume the position in question, a position which the patient more willingly observes, as pain is relieved from the capsule being then at its maximum capacity. But if the patient tries to walk when one limb is thus abducted, in order to avoid walking with the limbs separated, which would be very inconvenient, he approaches the sound to the diseased limb, that is to say,

*adducts* the sound limb, and then the lower extremities form, with the vertical axis of the pelvis and the trunk, a re-entrant angle towards the diseased side. But now the body is in a state of unstable equilibrium; and this, again, is corrected by flexion of the trunk on the pelvis towards the sound side, so as to bring the axis of the trunk in a line with that of the lower extremities. In the recumbent posture the same thing occurs also, for, unless the patient lies with the legs separated, the sound leg is approached to the other, and the resulting angular posture being very inconvenient, is as far as possible remedied by flexing the loins towards the sound side. M. Parise thus concludes that the pelvis itself is not deviate, that it is in fact a fixed point, on which the lower extremities are inclined laterally in one direction, and the trunk in the opposite direction. It is unnecessary to go in so much detail through the effects of the other deviations of the lower extremities. Each of them produces an inverse deviation of the trunk on the pelvis, in obedience to the law of equilibrium for preserving the line of gravity perpendicular, or nearly so, to the ground. Thus, the *flexion* of the thigh induces concavity of the loins from inclination of the trunk backwards, whence is caused apparent inclination of the pelvis forwards; and its *rotation outwards* induces torsion of the trunk, whence, as the position of the iliac spine is compared to the anterior plane of the trunk, the ilium on the affected side seems carried forward, while the apparent torsion of the pelvis will be in the reverse direction when the femur is *rotated inwards*. Again, at a certain advanced period of the disease, and sometimes at its commencement, the thigh becomes *adducted* and rotated inwards, continuing flexed. The cause of this *adduction*, M. Parise says, is, when it is primary, the existence of but a small quantity of fluid in the joint; and, when it is secondary, the gradual yielding of the capsule, but chiefly the escape of fluid from its erosion during the progress of the disease; but, be its cause what it may, when established, its effects are the reverse of those of *abduction*, viz. elevation of the affected side of the pelvis. The explanation of the results of inspecting and measuring both limbs in the first and second stage of morbus coxæ is obvious. *Apparent differences* to the *eye* result from our examining the lower extremities of two levers of equal length, whose upper extremities form unequal angles with the horizontal line connecting them. The cause of *apparent differences by measurement* has been already seen. It is equally obvious that, provided the malleoli are in contact, apparent shortening to the eye always accompanies apparent elongation by measurement, and vice versa. It might thence be naturally inferred that these two modes of examination would exactly correct each other; but such is not the fact, for it is curious that *lengthening to the eye* from abduction follows a rapid progression, and is not constantly proportional to the *shortening by measurement* of the same limb.

With respect to *real lengthening* of the limb in morbus coxæ, we shall merely notice such points in M. Parise's researches as contradict some pretty generally received ideas. It is, we believe, the prevalent opinion, founded both on theoretical considerations and on M. Fricke's experiments, that neither accumulation of fluid nor thickening of the structures within the hip-joint can protrude the head of the femur so as to cause elongation of the limb. Even M. Bonnet, who ascertained that liquid injected into the hip collects between the head of the femur and the bottom of the acetabulum, denies that any appreciable lengthening of the limb is caused thereby. M. Parise, however, and also M. Maisonneuve, found the limb very obviously elongated after injecting the joint, and from this and other experiments, such as introducing folds of lint into the acetabulum, &c., which it is unnecessary to dwell on, they conclude that accumulation of liquid or thickening of the structures within the joint must necessarily cause lengthening of the limb so long as the upper wall of the cotyloid cavity is intact. With respect, however, to increase in the size of the head of the femur causing lengthening of the limb, though M. Parise, on repeating Fricke's well-known experiments, found them erroneous, yet he concludes that in the rare cases in which the head of the bone is enlarged, lengthening of the limb cannot ensue, as in every known case of the kind the cotyloid cavity was proportionably enlarged also. As to the causes of *real shortening* of the limb without dislocation, M. Parise, on repeating Fricke's experiments, obtained results directly the reverse of those of that author, as artificial diminution of the head of the femur constantly produced shortening of the limb. The most important case, however, of true shortening without dislocation, is atrophy of the bones of the lower extremity, arising from arrest of development. This occurs very frequently, and, if overlooked, may occasion serious errors of diagnosis and prognosis; but error is avoided by measuring each limb comparatively from the great trochanter to the condyle of the femur, and from the head of the tibia to the outer ankle.

However clearly the causes of apparent alterations of the length of limb may be ascertained, still much difficulty often exists in determining the question practically. In the case of apparent elongation a satisfactory conclusion can indeed be obtained by placing the lower extremities symmetrically, and adopting the well-known method recommended by Sanson and A. Bérard; but, in apparent shortening, as the diseased limb is adducted, the limbs cannot be similarly posited, for though the sound limb may be brought to the same degree of adduction as the other, yet to do so it must be either more flexed or extended; and, if more flexed, it would seem shorter; if more extended, longer by measurement than it really is. M. Parise, therefore, concludes that we can only approximate to the truth in this case by taking the mean between measurements of the limb in those two positions; while M. Bonnet maintains that the difficulty can only be overcome by restoring the affected limb to its natural position, which often requires some time and the aid of suitable apparatus. As to the symptomatic value of alterations of the length of the limb, M. Parise concludes that *real shortening* from *atrophy of the bones* is a bad symptom, not merely because the limb must remain permanently shortened, but because it indicates a bad form of disease; that *apparent lengthening* to *the eye*, resulting from abduction and flexion, indicates a large collection of liquid in the joint, which may, by protruding the head of the femur, also occasion *real* lengthening; and that dislocation is imminent when this elongation, after having been decided, diminishes slightly, but motion continues as difficult and painful, and the great trochanter, previously prominent, becomes still more so. When *apparent shortening*, arising from adduction and flexion, exists at the commencement of morbus coxæ, the quantity of liquid in the joint is small, and the disease has probably commenced in the hard structures; when it succeeds apparent elongation, the capsule of the joint is probably perforated. Shortening *both* to the eye and by measurement, with prominence of the great trochanter, indicates dislocation; or, if the great trochanter is not prominent, erosion of the cartilages, or more or less destruction of the articular surfaces, according to the amount of shortening present.

M. Bonnet absolutely rejects resections in any of the large joints, even of the upper extremities. "They are," he says, "more fatal than amputations; and, after having been long considered one of the most brilliant conquests of modern surgery, they have now fallen into merited oblivion. I cannot even imagine any case in which resection of a large joint could be indicated." (Vol. i, p. 165.) We shall not stop to dispute or refute this sweeping condemnation, to which, indeed, we should not have adverted, were it not that, in addition to the resection of those joints, now pretty generally considered admissible, M. Bonino theoretically, and Mr. Fergusson practically (Med. Chirurg. for 1845, vol. xxviii), have recently inculcated the propriety of resecting the head of the femur in certain cases.

We believe that all the known cases of resection of the head of the femur are referred to in M. Bonino's and Mr. Fergusson's papers. M. Bonino enumerates twelve, including one doubtful case (Kluge's); and to these we may add Mr. Fergusson's own case, and one which he has been informed, by Mr. C. Hawkins, occurred in the practice of Sir B. Brodie. Excluding Kluge's doubtful case, thirteen remain, which may be thus classed. In two cases (Oppenheim, Seutin) the operation was performed in consequence of fracture of the upper extremity of the femur by gunshot wounds. Ten were cases of disease of the hip-joint. And in one (Textor), though probably, if not certainly, the patient, a child aged 7½ years, in the first instance laboured under disease of the joint, it was discovered after death that a fall at an early period of the affection had caused fracture of the neck of the femur, of the horizontal ramus of the pelvis, and of the ascending ramus of the ischium; and that those fractures, the existence of which was not suspected during life, had occasioned suppuration of the surrounding soft parts.

In appreciating the results of resection of the head of the femur in disease of the hip, the last case must clearly be left out of consideration. The two cases in which the operation was performed in consequence of gunshot wounds, do not directly bear on our present purpose, but we may say thus much respecting them, that, although they both terminated fatally, we are yet of opinion that in any similar case where the great vessels and nerves were intact and the soft parts not extensively injured, resection would be decidedly preferable to amputation at the hip, though the contrary opinion is advocated by M. Laillard (Relation Chirurg. du Siège de la citadelle d'Anvers). Of the ten patients on whom the operation was performed because of disease of the hip-joint, six recovered (White, Schlichting, Schmaltz, Heine, Vogel, Fergusson). All those patients were young, the two oldest, those of Schlichting and Mr. Fergusson, being both aged 14. The remaining four patients died (Hewson, Brodie,

Textor, two cases); they were all older than those in whom the operation succeeded, the youngest being, so far as we can make out, one of Textor's patients, aged 18.

The cause of death in the fatal cases seems to have been directly referrible to the operation but twice, that is to say, in one half of the fatal cases, and in one fifth of the whole number operated on for disease of the joint. Mr. Hewson's patient survived three months, and died from profuse suppuration, coexisting with, if not caused by, caries of the pelvis. One of Textor's patients survived fifty-four days, and died from sloughing over the sacrum, when the wound was nearly cicatrized and the upper extremity of the femur surrounded by newly deposited bone. A second of Textor's patients died in four days, and, though there was tubercular disease of the mesenteric glands, which, if known, would have contraindicated interference, yet death must be attributed to the operation, as was also, we are told, apparently the case with Sir B. Brodie's patient; but no particulars are given of this case, save that the patient was an adult; that the head of the bone was in the acetabulum at the time of the operation, and that the patient died a few days after it was performed.

The result of the operation in the successful cases was more favorable than might have been *a priori* anticipated. With respect to the patients of Schmaltz and Heine we are merely told that they recovered. A false joint formed and an useful limb was enjoyed by White's, Schlichting's, and Vogel's patients; and, in Mr. Fergusson's case, where 4½ inches of the bone, measuring round the curve of the neck, and shaft were removed, the limb, six months after the operation, was about 2½ inches shorter than its fellow, and a false articulation was evidently in process of formation at the hip.

The indication for the operation in disease of the hip-joint is the difficult part of the question. Most surgeons reject the operation, on the grounds that we cannot tell when the cotyloid cavity is implicated in the disease, in which case the operation must generally be useless. We believe that the disease most frequently, though by no means constantly, commences in the head of the femur, and that the bones of the pelvis become subsequently implicated; but, granting this, it only raises the presumption that the cotyloid cavity would be found healthy during the early period of the malady, when resection of the head of the femur would be utterly inadmissible. In all the cases of which we have any tolerably accurate account, in which the operation was performed, abscesses had opened externally, and the head of the femur could be felt denuded and isolate from the surrounding soft parts, the very stage of the disease in which the cotyloid cavity is usually implicated. But we may here mention that, though all Textor's patients died, yet the cotyloid cavity was found healthy in two of them; in the third it was diseased, and the actual cautery was applied after the surface of the bone was cut away. It is easy, however, to imagine a condition of things (we have seen it more than once) in which the diseased head of the femur, lying at the bottom of an ulcer or of a fistulous abcess, excites all the mischief of a foreign body, and in which its removal would seem to hold out the only chance of recovery. Under such circumstances, which existed in Mr. Fergusson's case, the operation may be justifiable, but it is one which, in the present state of our knowledge, cannot be indicated or contraindicated by fixed rules; everything must depend on the discretion and judgment of the surgeon, after carefully weighing all the circumstances of the particular case before him.

We scarcely feel it necessary to express any summary opinion as to the merits of M. Bonnet's work, after the very ample analysis we have given of its contents, the more especially as we have, for the most part, coupled that analysis with an expression of opinion respecting the author's views and practice. We shall therefore conclude this rather lengthened notice for the present; we say, for the present, inasmuch as we shall soon have occasion to return to the subject of diseases of the joints, when many matters not adverted to in this and the preceding article shall receive fitting consideration.

